# Opinion paper: artificial intelligence as a clinical ally in lung cancer detection—the case for Gleamer’s ChestView

**DOI:** 10.3389/fonc.2025.1673369

**Published:** 2025-10-20

**Authors:** Esteban Zavaleta-Monestel, Sebastián Arguedas-Chacón, Jeaustin Mora-Jiménez, Kevin Cruz-Mora, Abraham Rocha-Romero

**Affiliations:** ^1^ Health Research Department, Clínica Bíblica, San José, Costa Rica; ^2^ Pharmacy Department, Clínica Bíblica, San José, Costa Rica; ^3^ Facultad de Farmacia, Universidad de Costa Rica, San José, Costa Rica

**Keywords:** chest X-ray, lung cancer detection, computer-aided detection, diagnostic accuracy, clinical implementation

## Introduction

The global incidence of lung cancer varies depending on the source and year of estimation. However, recent and widely recognized data estimate that in 2022, approximately 2.5 million new cases of lung cancer were diagnosed worldwide, accounting for 12.4% of all cancer diagnoses. Moreover, it was responsible for about 18.7% of cancer-related deaths, making it the leading cause of cancer mortality globally. When excluding non-melanoma skin cancer and melanoma, lung cancer also ranked first in global incidence for that year (1, 2).

There are multiple subtypes of lung cancer; nevertheless, they all share a common characteristic, the rapid progression from early to advanced stages. Therefore, timely diagnosis is essential to initiate treatment as soon as possible and prevent disease advancement (3). In this context, chest imaging plays a crucial role by allowing the early detection of pulmonary nodules. Chest X-ray (CXR) is the primary imaging tool for detecting, assessing, and monitoring cancers such as lung cancer (4).

Although recent studies show that CXR for detecting lung cancer in the general population and symptomatic patients has a sensitivity ranging from 60% to 76%, its specificity is high at 94–95%. This means that about a quarter of early-stage lung cancer cases go undetected, possibly delaying diagnosis and allowing disease progression. Additionally, a negative diagnosis does not reliably exclude the presence of lung cancer (5).

These limitations have led to the development of computer-aided detection (CAD) systems based on artificial intelligence (AI) to enhance the automated analysis of CXR. Among these solutions is ChestView, developed by the company Gleamer, which employs deep learning algorithms trained with millions of clinical images. This tool does not aim to replace radiologists but rather to act as a diagnostic copilot, offering a second automated reading that facilitates early detection of findings suggestive of lung cancer (6).

## Features of Gleamer’s ChestView

ChestView, developed by the French company Gleamer, is a computer-aided detection (CAD) solution based on artificial intelligence specifically designed for chest X-ray analysis. This tool is CE certified under the European Medical Device Regulation (EU MDR, class IIa), endorsing its clinical use in hospital settings. ChestView uses a deep convolutional neural network (CNN) based on the Detectron2 architecture, which is an open-source framework created by Facebook AI Research for object detection in medical images. The algorithm is trained to identify five common thoracic abnormalities: pneumothorax, pleural effusion, consolidation, mediastinal or hilar mass, and pulmonary nodule (6).

## Current evidence of ChestView as an AI clinical support tool

Artificial intelligence has recently been implemented to support the detection of thoracic diseases in chest radiographs. Bennani et al. (2023) evaluated its utility through a retrospective study of over 500 patient X-rays, focusing on identifying anomalies such as pneumothorax, pleural effusion, consolidation, mediastinal and hilar masses, and pulmonary nodules (7).

Key findings included an average absolute increase of 12% in sensitivity for detecting pulmonary nodules and a significant reduction in average reading time of 81 seconds without AI to 56 seconds with AI (a 31% decrease; P < 0.001) (7).

Furthermore, for pulmonary consolidations, AI achieved an AUC of 0.93, significantly outperforming the average performance of radiologists without AI assistance (AUC 0.71). These results demonstrate that AI not only supports human readers but also has the potential to elevate diagnostic standards in clinical practice (7).

In another study, Chassagnon et al. (2023) included eight radiology residents to evaluate whether a computer-aided detection (CADe) system could serve as a learning tool for interpreting CXR. Key findings showed improved sensitivity (53% vs. 43%; p < 0.001), specificity (94% vs. 90%; p < 0.001), and accuracy (86% vs. 81%; p < 0.001) during the intervention (8).

**Figure 1:**
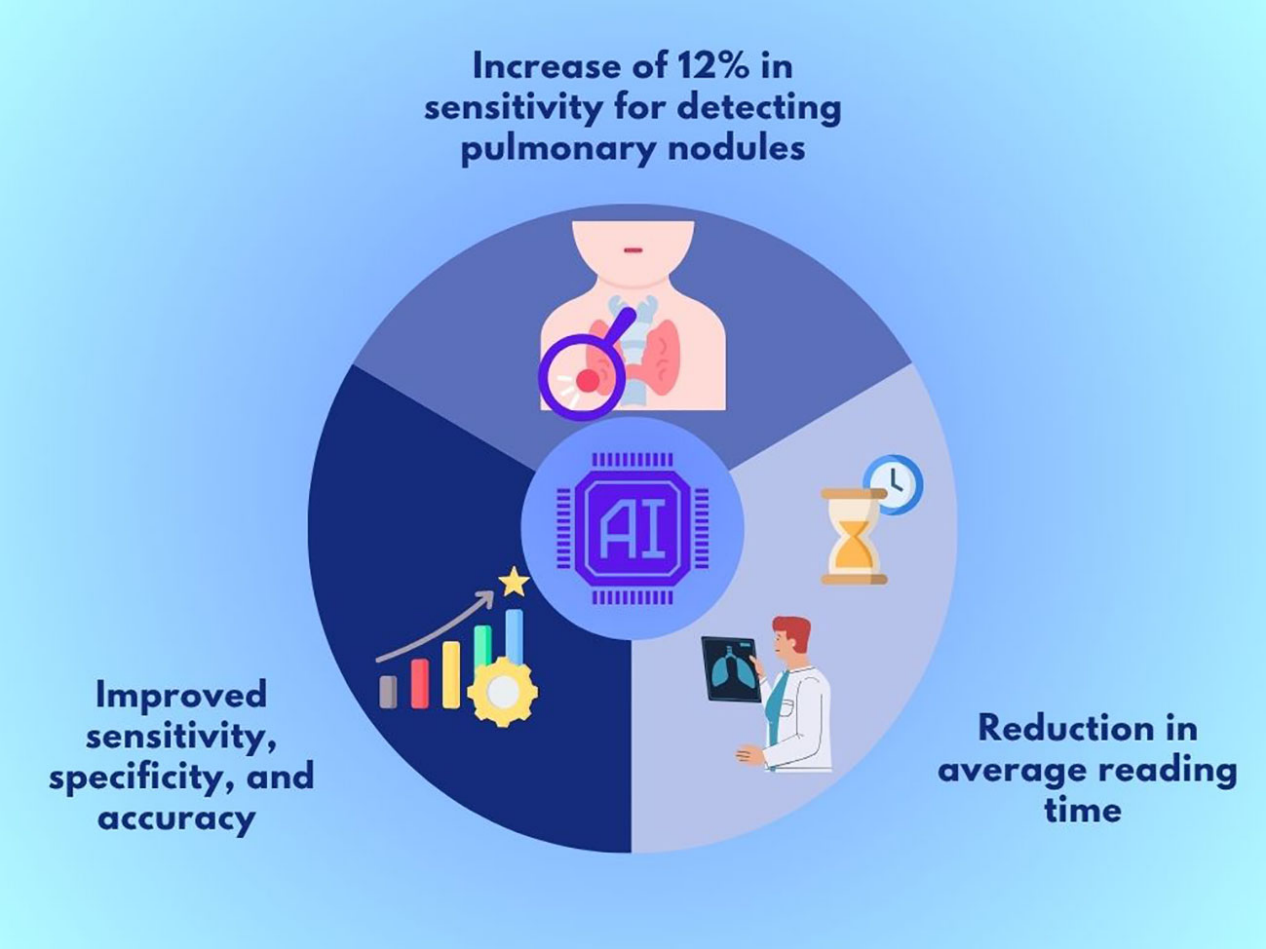
Key outcomes of ChestView AI support in chest X-rays.

The key outcomes of ChestView AI support are summarized in **Figure 1**, which illustrates improvements in sensitivity, specificity, diagnostic accuracy, and interpretation time.

## Barriers and considerations for clinical adoption

In addition to the methodological considerations mentioned, other factors hinder the safe and effective implementation of AI in daily clinical practice. One such factor is the variable quality of images used in both training and application of models. In real-world contexts, images may contain artifacts, suboptimal positioning, or technical differences that do not present in ideal datasets, negatively affecting the performance of CADe systems (8).

A lack of heterogeneity in training data limits the generalizability of algorithms. Many models have been developed using databases from specific centers, preventing results from being extrapolated to more diverse clinical populations or different technical environments. This lack of representativeness may introduce bias and reduce the applicability of the tool in routine settings (8).

Another consideration is the computational and operational demands associated with implementing these systems in clinical practice. While model training is performed externally by the developer, their clinical use still requires adequate technological infrastructure, which may represent a barrier, particularly in resource-limited institutions (8).

Lastly, although regulatory pathways such as FDA clearance in the US and the MDR in the EU exist, the absence of harmonized and internationally standardized frameworks tailored for AI in radiology remains a critical limitation. This gap creates legal and ethical uncertainty regarding adaptive algorithms, post-market validation, and professional responsibility, complicating their formal integration as diagnostic or educational support tools (8).

## Discussion

Gleamer’s ChestView represents a scientifically tangible example of the positive impact AI tools can have on early lung cancer detection using chest radiographs. Current evidence suggests that its use may positively influence diagnostic sensitivity, improve image interpretation accuracy, and optimize diagnostic timing. This not only represents a direct clinical benefit but also an opportunity to align and leverage technological advances for patient health (9).

However, its effective implementation in clinical practice still faces significant limitations, such as the need for advanced technological infrastructure, operational costs, and the lack of standardized regulations to govern its use. These aspects must be considered to avoid diagnostic biases, ensure patient safety, and maximize the added value of these technologies (10).

In conclusion, ChestView has the potential to become a reliable diagnostic tool for medical personnel, especially in clinical contexts where time for accurate interpretation is limited. Its successful future integration will depend on ongoing clinical studies, multicenter validation, and the development of regulatory standards supporting its safe, ethical, and effective use.
